# Duodenal diverticulization as treatment of complex duodeno-pancreatic lesions: Case report

**DOI:** 10.1016/j.ijscr.2019.12.014

**Published:** 2019-12-17

**Authors:** Caroline Petersen da Costa Ferreira, Natyele Soares Lima, Maria Carolina Galli Mortati, Mauricio Alves Ribeiro, Mohamed Ibrahim Ali Taha, Jacqueline Arantes Giannini Perlingeiro, Jose Cesar Assef

**Affiliations:** a Emergency Service of the Irmandade da Santa Casa de Misericórdia de São Paulo (ISCMSP), São Paulo, SP, Brazil; b General Surgical Residency of the Irmandade da Santa Casa de Misericórdia de São Paulo (ISCMSP), São Paulo, SP, Brazil

**Keywords:** DI, Duodenal injuries, PI, pancreatic injuries, AAST, American Association to the Surgery of Trauma, SAMU, Mobile Emergency Service, ISCMSP, Brotherhood of Santa Casa de São Paulo, BP, Blood Pressure, HR, Heart Rate, IU, International Units, PO, post-operative, ICU, Intensive Care Unit, ERCP, Endoscopic Retrograde Cholangiopancreatography, Duodenal and pancreatic trauma, Abdominal trauma, Duodenal diverticulization, Case report

## Abstract

**Introduction:**

Duodenal and pancreatic lesions are uncommon, but severe and responsible for high incidence in morbidity and mortality. Differences between the mechanisms of trauma, the severity of lesions and the time between trauma, diagnosis and treatment influence the evolution of the case.

**Presentation of case:**

We report a case of a 20-year-old patient with several lesions in stomach, duodenum, pancreas and jejunum due to three gunshots treated at our service. Duodenal diverticulalization was used on treatment of complex duodeno-pancreatic lesions. The patient presented good evolution, with discharge conditions in the 10th PO.

**Discussion:**

We discussed the positives and negatives of this technique, with the approval of the Ethics Committee number 13736519.8.0000.5479.

**Conclusion:**

The duodenal diverticulization leads to an irreversible change to the food transit. However, this is a feasible bypass option in cases of high chances of fistula and scar stenosis complex duodenal injury, particularly in the context of associated gastric injury.

## Highlights

• Duodenal injuries (DI) and pancreatic injuries (PI) are uncommon, but can be life-threatening.
• Severe duodenal injuries require more complex surgical procedures, including duodenal diverticulization.
• The duodenal diverticulization is a feasible option in cases of complex duodenal injury with high chances of fistula and scar stenosis.

## 1 Introduction

Duodenal injuries (DI) and pancreatic injuries (PI) are of comparatively uncommon occurrence among abdominal traumas, accounting for 3,7–5 %, and are associated with significant mortality and morbidity [1]. Factors as trauma mechanism, associated injuries and time between trauma and treatment are determinative of the prognosis.

Most of the times, DI result from penetrating abdominal trauma. In 20 % of the cases, there are associated PI that worse the prognosis significantly [2]. The high rates of complications related to this kind of injury are due to the delay in diagnosis and/or unnoticed injuries, resulting from their insidious nature, in addition to their anatomic retroperitoneal location.

Pancreatic lesions are rarer than DI and have higher morbidity and mortality, since the diagnosis is, usually, late. Because of their anatomical proximity to other organs, they are rarely an isolated injury. Isolated lesions of the pancreas occur in 2 % of abdominal traumas [3].

American Association to the Surgery of Trauma (AAST) in agreement with the Organ Injury Scale Committee (AAST-OIS) divided injuries into grades [4] (Table 1, Table 2).

**Table 1:** Duodenal Injury Scale.

Grade I	**Hematoma**: Involves only one duodenal portion **Laceration**: Partial laceration, without perforation.
Grade II	**Hematoma**: Involves more than one portion. **Laceration**: Less than 50% disruption of the circumference
Grade III	**Laceration**: 50%–75% disruption of D2 circumference 50 %–100 % disruption of D1, D3 and D4 circumference
Grade IV	**Laceration**: More than 75% disruption of D2 circumference Involves the ampulla or the distal portion of the common bile duct
Grade V	**Laceration**: Massive disruption of the duodenopancreatic complex. **Vascular**: Duodenal devascularization

**Table 2:** Pancreatic Injuries according to the AAST.

Grade I	**Hematoma**:Minor contusion without ductal lesion **Laceration**: Superficial laceration without ductal lesion
Grade II	**Hematoma**: Greater contusion without ductal lesion or tissue loss **Laceration**: Larger laceration without ductal lesion or tissue loss
Grade III	**Laceration**: Distal transection or parenchymal lesion with ductal lesion
Grade IV	**Laceration**: Proximal transection (to the right of the superior mesenteric vein) or parenchymal lesion involving the ampulla
Grade V	**Laceration**: Mass destruction of the head of the pancreas

According to AAST, grades III, IV and V duodenal injuries are serious. Grades I and II injuries account for 70–80% of the DI and are susceptible to primary repair. Grades III to V injuries are less frequent and require more complex surgical procedures for their definite treatment, including duodenal diverticulization, pyloric exclusion, duodeno-jejunal anastomosis, duodenal-duodenal anastomosis or more exceptionally duodenopancreatectomy – conducted only in cases of uncontrollable pancreas hemorrhage or injury associated to bile or pancreatic ducts.

Surgical intervention in pancreatic lesions occurs in lesions > grade III. Grade I and II lesions without ductal lesion, resulting from closed abdominal trauma with stable patients, may be treated conservatively [3].

We report the case of a patient with DI, PI and other associated injuries due to gunshot wound; with the approval of the Ethics Committee number 13736519.8.0000.5479.The work has been reported in line with the SCARE criteria [5]. Complex duodenal lesions are uncommon, and most surgeons have little experience in this matter. Thus, the didactic presentation of a case may contribute to the proper use of the surgical techniques.

## 2 Case report

The Mobile Emergency Service (SAMU) brought a 20-year-old caucasian male victim of gunshot wounds to the Emergency Service of our Institution. On the scene, he was hemodynamically unstable, with Blood Pressure (BP): 90/60 mmHg and a Heart Rate (HR):130 bpm. The transference to the Emergency Service took 30 min and the pre hospital staff provided 500 ml of crystalloid solution. He denied illness and reported drug use (marijuana).

At admission, he remained hemodynamically unstable. Examination of the respiratory system revealed diminished breath sounds throughout the right hemithorax, without adventitious sounds with ipsilateral subcutaneous emphysema. The BP upon admission was 100 × 40 mmHg and HR: 116 bpm.

**Fig. 1:**
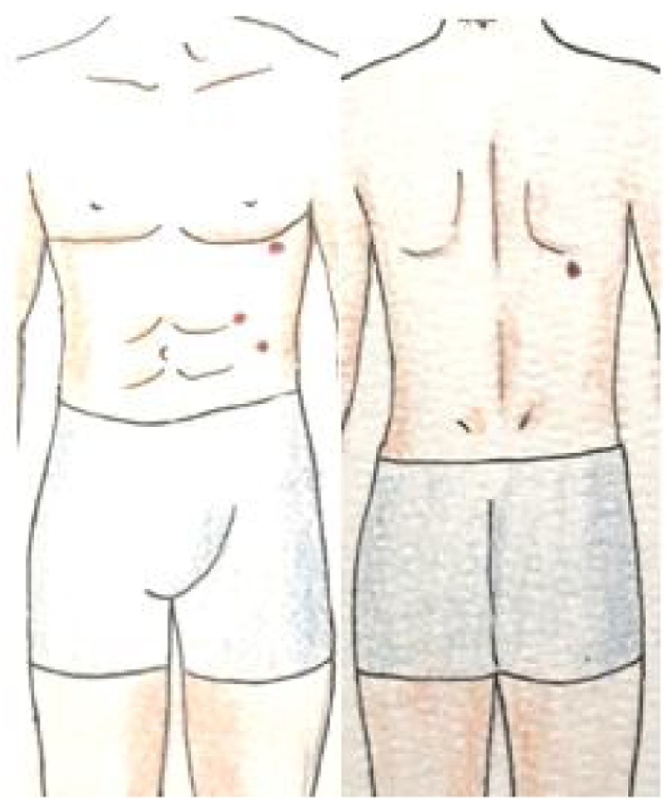
Entry and exit holes location.

There were three gunshot entrance wounds with abrasion rings: one in the right thoracoabdominal transition and two other periumbilical ones (Fig. 1). He also had a back exit wound. He received medical care according to the protocol recommended by ATLS (Advanced Trauma Life Support). After placing an oxygen mask (10 liters/minute), the patient was monitored. Two peripheral large venous accesses were punctured, and he received 500 milliliters of physiological solution (SF0.9 %). The laboratory tests (blood typing, hemoglobin, and blood reserve) were performed. In addition, a 38 French intercostal drain was inserted on fifth intercostal space in the right thorax, because of an hemothorax in the right, connected to an underwater seal bottle that immediately began to bubble. Three hundred milliliters of blood poured into the bottle, causing a subsequent stabilization of BP and HR. A chest X-ray, performed in the trauma room, provided a good location of the chest drain.

**Fig. 2:**
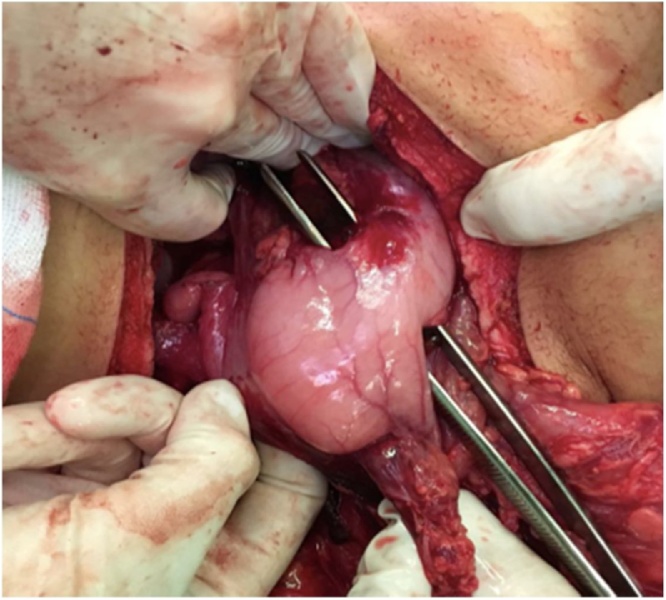
Penetrating lesions in small anterior wall of the stomach.

**Fig. 3:**
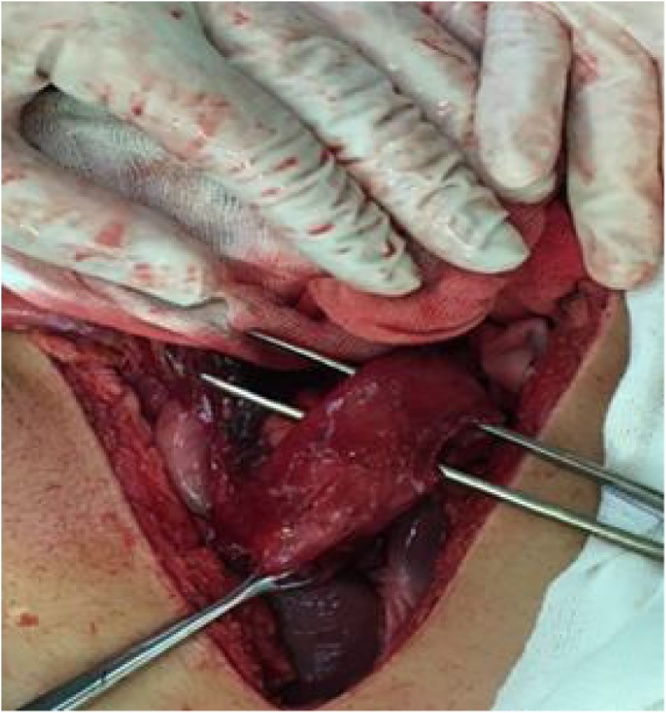
Penetrating lesion in second duodenal portion -greater than 50 % of organ diameter.

**Fig. 4:**
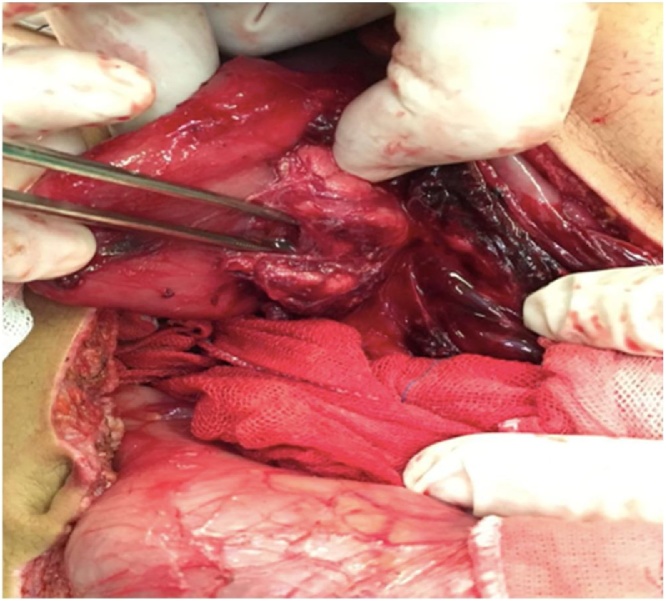
Penetrating lesion in the second duodenal portion greater than 50 % of the diameter of the organ associated with pancreatic lesion.

The patient was taken to the operating room and placed on the operating table in the supine position for an exploratory laparotomy. During the procedure, it were identified several lesions in stomach, duodenum, pancreas and jejunum. There were three stomach injuries: two on anterior wall (Fig. 2) and other one on the posterior wall; three duodenal injuries: one in the first duodenal portion, other penetrating one on second duodenal portion (Fig. 3); a grade II head pancreatic injury (Fig. 4) and two penetrating jejunum injuries.

The first duodenal portion injury was Grade II and the second duodenal portion one was Grade III.

**Fig. 5:**
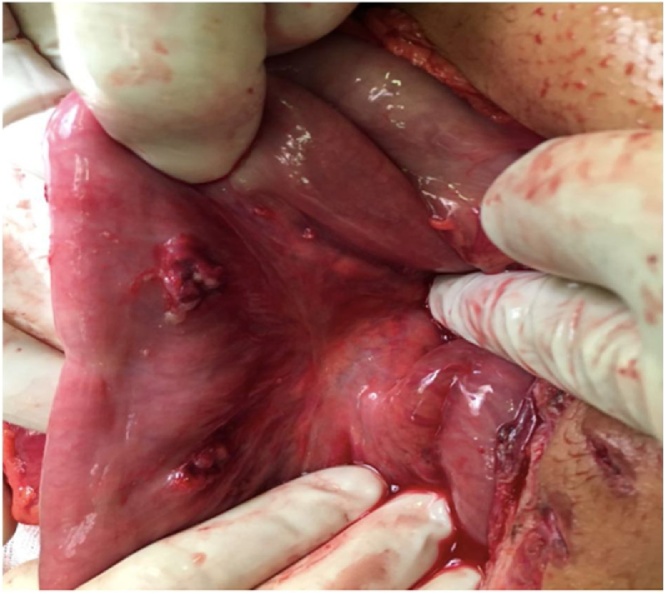
Small Intestine Injuries.

The small intestine transfixing lesions were six centimeters apart from each other at 10 cm from the Treitz angle (Fig. 5).

**Fig. 6:**
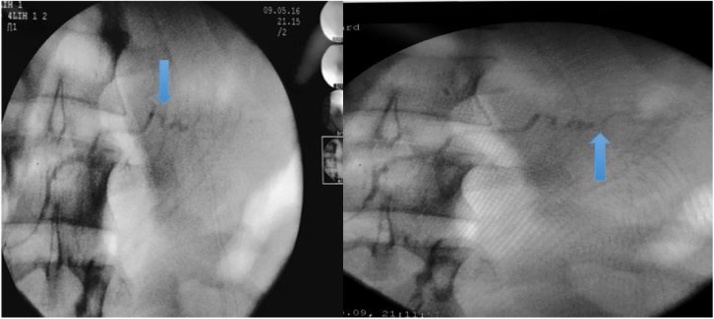
Pancreatography.

At this time, the patient was hemodynamically stable -following satisfactory response to volume replacement- with no acidosis on arterial blood gas analysis. Then, it was chosen to perform definitive surgery rather than damage control: debridement and primary suturing of the duodenal injuries; bypass through duodenal diverticulization - partial gastrectomy with Billroth II reconstruction and enterectomy with entero-enteroanastomosis and full drainage of the cavity. Catheterization of the duodenal papilla was also conducted, with pancreatography, excluding Wirsung duct injury (Fig. 6).

The anesthetic time was four hours and forty minutes. The patient received two Units (IU) of red blood cell concentrate, 7300 ml of crystalloid and 500 ml of colloid, with 900 ml diuresis in the intra-operative period.

The patient presented good evolution, being discharged from the ICU on the second post-operative (PO) day, the thorax drain being removed in the third PO. The patient progresses with low output pancreatic fistula resolved in the sixth PO, receiving oral diet the next day, with discharge conditions in the 10^th^ PO. The patient returned for an outpatient visit after 15 days with no complaints.

## 3 Discussion

Although being poorly frequent, the duodenum and pancreas traumatic injuries represent a challenge to the urgency and trauma surgeon. The anatomy of the pancreatic-duodenal topography and the proximity of the other organs promote injuries of complex nature, requiring technical refinement and anatomic knowledge by the surgeon [6].

According to the literature the vast majority of the pancreaticoduodenal injuries are penetrating abdominal injuries in 75 % of the cases, firearm injuries for example and in 25 % of cases are blunt trauma (traffic accidents, sport injuries) [5]. Ahmad et al. [8] related 3 case reports of isolated duodenal injuries after blunt trauma. In all cases, the laboratory, image and physical exams initially were unremarkable. Laparotomy was planned due the presence of minimal amount of free fluid in the peritoneal cavity or vital sings deterioration. During the surgery, they could observe the duodenal laceration after complet kocherization of the duodenum.

Regarding duodenal injuries, second segment (36 %) is the most prone to traumatic injuries, followed by third segment with 18 %, fourth segment with 15 %, and first segment with 13 % [7,9]. In Bogotá, in its turn, Timaran et al. [10] treated in a four-year period 152 patients with penetrating trauma (Injure by firearm projectile in 53 % and injury by cold weapon – in 47 %).

The early diagnosis, the choice of the appropriate surgical technique to identify and repair the injury, and the correct treatment of the complications are morbimortality-reducing factors.

In case of penetrating trauma, the diagnosis is generally earlier. In the intra-operative period, a detailed inventory of the abdominal cavity is important, and in case of suspected duodenal injury, the Kocher maneuver, the medial mobilization of the hepatic flexure of the colon (Cattell-Braasch) and the medial elevation of the duodenum and the pancreas head must be conducted in order to search for lesions in the posterior wall. The exposure of the forth duodenal portion is conducted by the medial displacement of the cecum to the mesenteric root and the Treitz angle, or by sectioning the duodenojejunal ligament, in search for injuries [[10], [11], [12]]. Care must be taken in case of large retroperitoneum hematomas due to the presence of associated vascular injury that may draw the surgeon’s attention away, causing an injury to be unnoticed.

If the topographic location of the duodeno-pancreatic complex, on the one hand, provides certain protection, with low incidence of injuries (3–5%), on the other hand, impair the early diagnosis of the injury [10,12].

In a review by Silveira et al., 2009 studied 131 patients with abdominal trauma report. The results of the study indicated that 64.9 % were gunshot wounds, of these, the majority reached was male. This study verified that the overall morbidity was 64.9 %, with 29 % of complications directly related to the pancreas, such as fistulas and bleeding. Being the highest morbidity and mortality in patients with complex lesions (grades IV and V) of the pancreas when compared to less severe lesions (grades I and II) [13].

Lucas and Ledgerwood [14], in 1975, showed that the delayed diagnosis and surgical treatment of the duodenum trauma were responsible for the high morbimortality indexes. These authors noted a mortality of 14 % on patients submitted to surgery within the first 24 h after the trauma, and 40 % when the surgery was conducted following such period. Snyder et al. [15] reported a mortality of 50 % in patients lately submitted to surgery, with an incidence of fistula of 50 % in the survivors.

The Grade II and III injuries are the most frequent injuries in the case-by-case analysis, particular when the penetrating traumas are prevalent [9,16].

The duodenal trauma is generally associated with a high incidence of injuries in other abdominal organs and viscera [10,17,18].

The technique used in the surgical treatment of the duodenal traumas is discussible due to the great variety of therapeutic options resulting from a number of variables that must be considered in such circumstances. Most (70–85%) of the duodenal injuries is candidate to simple procedures such as: debridement of the devitalized tissue and primary repair, or resection and anastomosis plus drainage [12,17,18]. Patients with extensive duodenal injuries can be candidates to more complex procedures, such as, duodenal diverticulization, pyloric exclusion or duodenopancreatectomy.

The segregation between the hemodynamically and metabolically stable patients or those who reached stability following resuscitation and those who are unstable is crucial. For those who do not recover the physiological integrity, the surgical procedure to be chosen is the one that limits the cavity contamination, even if it is not the definite procedure. Resections and reconstructions taking longer times are prohibited in hypovolemic, acidotic, hypothermic or coagulopathy patients [6]. The surgeon’s instinct is to treat every injury using the best surgical technique, however, in severely ill patients, brevity is essential and physiology prevails over anatomy [19]. For the other patients, it is convenient to choose a therapeutic option with definite resolution.

**Fig. 7:**
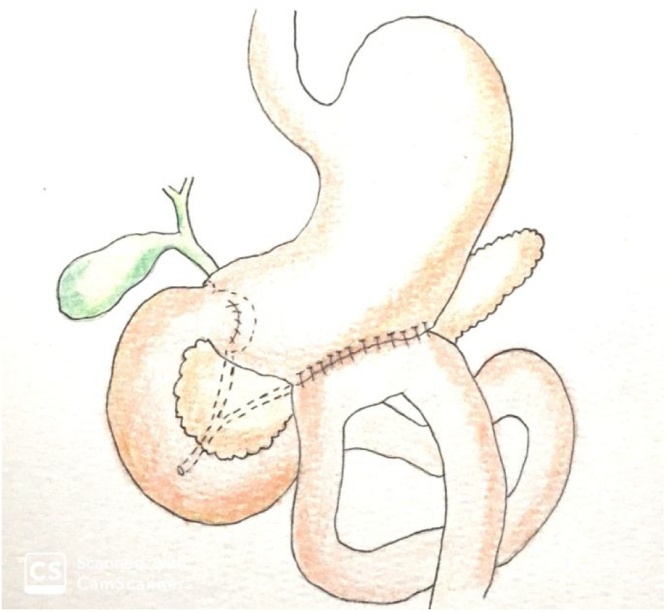
Pyloric exclusion.

The pyloric exclusion (Fig. 7) consists in suturing the duodenal injury, closing the pylorus with absorbable yarn, through gastrostomy, followed by gastrojejunostomy in the gastrostomy site, without troncular vagotomy, without gastrectomy and without biliary bypass. This diverts the gastric flow away from the duodenum for several weeks while the duodeno-pancreatic injuries are healed. The pylorus eventually opens (around 1–2 months after) and gastrojejunostomy functionally closes [20].

In duodenopancreactectomy – Whipple’s surgery – the distal’s stomach, the pancreas’ head and the entire duodenum, initial portion of the jejunum, gallbladder, choledochus and pancreas’ head are resected. In very extensive proximal duodenum and pancreatic head injuries, the destruction of the ampulla, of the pancreatic duct or the bile duct may prevent reconstruction, requiring such an approach. However, due to the magnitude of the surgery, this is an exceptional procedure on trauma.

Proposed by Benne in 1968, after having performed it in 16 patients, duodenal diverticulization consisted in the primary closure of the duodenal injuries, antrectomy, termino-lateral gastrojejunostomy and lateral-tube duodenostomy. In case of PI or DI close to Vater’s ampulla or the extra-pancreatic portion of the common bile duct, the orientation was the drainage of the biliary tract. Vagotomy was recommended once it decreases the enzymes rich in pancreatic juice, and may decrease the possibility of erosive gastritis in the immediate post-operative period, and may prevent the appearance of gastric ulcer [21]. Subsequently, in 1974, Benne and colleagues published 34 additional cases of duodenal diverticulization with 16 % of total mortality [22].

**Fig. 8:**
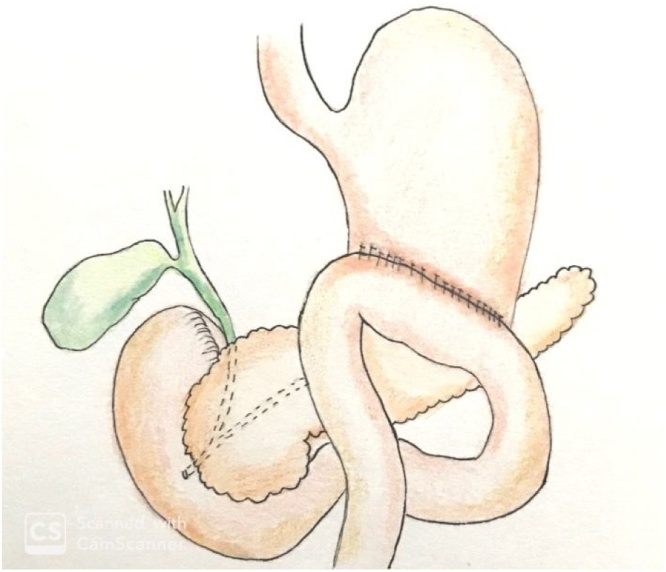
Duodenal diverticulization.

In the case described herein, the primary suturing of the duodenal injuries, plus a partial gastrectomy with gastrojejunostomy, intra-operative pancreatography and drainage of the cavity were performed (Fig. 8). We believe that in case of biliary fistula, the duodenum is not in the food transit and it would by a guided fistula. Nowadays, the ease of endoscopic retrograde cholangiopancreatography (ERCP) and the possibility of placing biliary prostheses for the treatment of fistulas allows us to be somewhat more conservative intraoperatively. The non-conduction of duodenostomy and vagotomy reduce the procedure time and morbidity.

Some factor may guide the surgeon to indicate one of such procedures: the blunt trauma or the one caused by high-energy projectile, the delay in treatment of more than 24 h, grade ≥ III injuries, pancreas- or extrahepatic bile duct-associated injury, or devascularization of the duodenum [23].

Velmahos et al. [23] indicated complex repairs in 32 DI cases (out of a total of 145 patients), and concluded that such injuries are frequently associated with other fatal injuries, and that the liberal use of major procedures in severe duodenal injuries prevents the morbidity directly resulting from such injuries.

The morbidity rate resulting from duodenal injury ranges between 30 and 100 %, although only one third of such finding is directly attributed to duodenal injury [15,17,18,24]. Among the most common complications are the dehiscence and fistula from anastomosis (around 7 % of the cases, reaching 16 % in some studies), pancreatitis, intra-abdominal abscess and pulmonary complications, which are directly or indirectly responsible for the development of sepsis and multiple organ failure. Cogbill et al. [16] observed that post-duodenal trauma morbidity is more dependent on the severity of the associated abdominal injuries than on the extension of the duodenal injury.

Mortality ranges from 5.3–30%, however, the mortality directly attributed to duodenal injury is around 10 % and the death resulting from the injury repair complications generally occurs within one to two weeks following trauma [12,17,18].

In general, the surgical approach of the duodenal trauma is intended to control hemorrhage, followed by the care to limit contamination from the digestive content leakage and identification of pancreatic and bile duct injuries.

## 4 Conclusion

The duodenal diverticulization leads to an irreversible anatomical change to the food transit. However, this is a feasible bypass option in cases of high chances of fistula and scar stenosis complex duodenal injury, particularly in the context of associated gastric injury.

## Sources of funding

At our own expenses. There are no sources of funding.

## Ethical approval

Santa Casa de São Paulo Ethics and Research Committee in 05/11/2018.

Reference number: 13736519.8.0000.5479.

The study was accepted and approved by Ethics and Research Committee of Santa Casa de São Paulo (CAAE: 13736519.8.0000.5479).

## Consent

Written informed consent was obtained from the patient for publication of this case report and accompanying images. A copy of the written consent is available for review by the Editor-in-Chief of this journal on request.

## Author contribution

Ribeiro M.A: Conceptualization; Editing and Visualization.

Lima N.S: Data curation.

Taha M.: Formal analysis, surgeon during the surgical procedure.

Ferreira CPC: Surgeon during the surgical procedure, Writing – Review & Editing.

Mortati, CG: Investigation, Writing original Draft, Perlingeiro J.A.G: Supervision and Project Administration, Postoperative care responsible.

Assef: Supervision, Review, Head of Emergence Service.

## Registration of research studies

Case reports that are not first-in-man study do not need Registration of Research Studies.

Already approved in Ethics Committee.

## Guarantor

Caroline Petersen da Costa Ferreira.

## Provenance and peer review

Not commissioned, externally peer-reviewed

## Declaration of Competing Interest

There are no conflicts of interest relevant to this article.
